# Robust Epileptic Seizure Detection Using Long Short-Term Memory and Feature Fusion of Compressed Time–Frequency EEG Images

**DOI:** 10.3390/s23239572

**Published:** 2023-12-02

**Authors:** Shafi Ullah Khan, Sana Ullah Jan, Insoo Koo

**Affiliations:** 1 Department of Electrical Electronic and Computer Engineering, University of Ulsan, Ulsan 44610, Republic of Korea; 2 School of Computing, Edinburgh Napier University, Edinburgh EH10 5DT, UK; s.jan@napier.ac.uk

**Keywords:** artificial intelligence, EEG, seizure detection, continues wavelet transform, hybrid features

## Abstract

Epilepsy is a prevalent neurological disorder with considerable risks, including physical impairment and irreversible brain damage from seizures. Given these challenges, the urgency for prompt and accurate seizure detection cannot be overstated. Traditionally, experts have relied on manual EEG signal analyses for seizure detection, which is labor-intensive and prone to human error. Recognizing this limitation, the rise in deep learning methods has been heralded as a promising avenue, offering more refined diagnostic precision. On the other hand, the prevailing challenge in many models is their constrained emphasis on specific domains, potentially diminishing their robustness and precision in complex real-world environments. This paper presents a novel model that seamlessly integrates the salient features from the time–frequency domain along with pivotal statistical attributes derived from EEG signals. This fusion process involves the integration of essential statistics, including the mean, median, and variance, combined with the rich data from compressed time–frequency (CWT) images processed using autoencoders. This multidimensional feature set provides a robust foundation for subsequent analytic steps. A long short-term memory (LSTM) network, meticulously optimized for the renowned Bonn Epilepsy dataset, was used to enhance the capability of the proposed model. Preliminary evaluations underscore the prowess of the proposed model: a remarkable 100% accuracy in most of the binary classifications, exceeding 95% accuracy in three-class and four-class challenges, and a commendable rate, exceeding 93.5% for the five-class classification.

## 1. Introduction

Approximately 1% of the global population is affected by epilepsy [1]. This condition poses significant challenges that can even be life-threatening for those affected. Among these patients, one-third do not respond to medications and need physical interventions [2,3]. Epileptic seizures are characterized by swift and abnormal fluctuations in the electrical patterns of the brain [4]. In severe cases, they can cause the entire body to become unresponsive [5]. Electroencephalogram (EEG) signals have been the fundamental reference for detecting epileptic seizures, helping to identify the seizure origin, and facilitating the treatment of the affected brain tissues through medication and surgical procedures [6]. EEG signals contain significant features that detail both regular and irregular brain activities, particularly epileptic seizures. In addition, high-temporal-resolution EEG data from the scalp, spanning multiple input channels, can be acquired through distributed continuous sensing techniques [7]. Traditionally, diagnosing epilepsy through visual analysis of EEG recordings, both clinically and conventionally, is labor-intensive and prone to error, with varying consistency among experts, because of its heavy reliance on human expertise and skill [8,9].

Many EEG automatic seizure detection systems struggle with real-time specificity and sensitivity, making them less suitable for clinical applications. There is a pressing need for an advanced computer-aided system that can efficiently assist neurologists in detecting epileptic seizures, ultimately reducing the time spent analyzing extensive EEG recordings [10]. In areas with a scarcity of neurologists, the excessive dependence on human expertise can increase the costs and cause delays in treating epilepsy. Tackling these issues is essential to guarantee affordable epilepsy care in low-to-middle-income regions, particularly in isolated locations with restricted access to skilled professionals and advanced facilities. Improving access to automated seizure detection using EEG signals has been studied extensively to mitigate this issue [11].

Machine learning is used widely to detect diseases automatically from biomedical signals, such as ECG and EEG. For example, a previous study [12] used two distinct features to detect epileptic seizures: fractal-based nonlinear features and entropy-based features. These features were inputted into two machine learning classifiers: Support Vector Machine (SVM) and K-Nearest Neighbor (KNN). The classifiers were trained and tested on the Bonn Epilepsy database. This database comprises five distinct classes: Set S, Set F, Set N, Set O, and Set Z. Set S represents seizure activity typically observed in epileptic patients. Both Set F and Set N denote seizure-free states in the epileptic class, Set O is associated with a normal, non-epileptic state where the subject’s eyes are closed, while Set Z corresponds to the normal state with the subject’s eyes open. In their evaluation, they considered binary (e.g., Z–S, O–S, and N–S) and the three-class detection problem (ZO–NF–S). In addition, another study [13] introduced a framework that integrates fuzzy-based methods and conventional machine-learning techniques to identify epileptic EEG samples in binary classification problems. A limited set of features and linear (using the Naïve Bayes classifier) and nonlinear (using the K-Nearest Neighbor classifier) approaches were applied to classify the EEG samples [14]. Binary classification tasks were involved in classifying various classes, i.e., Z–S, O–S, N–S, F–S, ZO–S, and ZN–E. Similarly, another study [15] used the statistical features and classified with SVM (AdaBoost Least-Square SVM). The resulting accuracy for the binary FNOZ-S classification problem in the Bonn dataset was 99%. In particular, none of these authors extended the evaluation of their proposed methods to include multi-class classifications.

Beyond traditional machine learning techniques, various deep learning architectures have been introduced to detect epileptic seizures in the EEG data. A previous study [16] utilized deep learning approaches to extract the important features from EEG data. In particular, a Convolutional Neural Network (CNN) was implemented for the differentiation tasks among normal, preictal, and seizure classes. The author of [17] introduced an experimental and methodological approach that mapped microscale local network dynamics with high spatiotemporal resolution and employed a quantitative analysis framework to elucidate the dynamics of seizure initiation and progression in vivo. In addition, the discrete wavelet transform (DWT) was used for feature extraction from the EEG data [18]. A combination of genetic algorithm and artificial neural network (ANN) and the Support Vector Machine (SVM) classifiers were used to address binary and three-class classification challenges in the Bonn Epilepsy database.

Many seizure detection methods concentrate on specific domains, such as utilizing time–frequency domain methods, i.e., continuous wavelet transform (CWT), time domain, frequency domain, and statistical attributes [19,20,21,22]. Unlike the other methods, the proposed epileptic detection model innovatively combines the best of these attributes. A comprehensive set of important features is obtained by leveraging the complex insights from the statistical domain that is characterized by rich features, such as the mean, median, variance, skewness, and kurtosis, with the compressed time–frequency domain images (CWT Images) processed through an autoencoder. This hybrid integration of Convolutional Autoencoder (CAE) latent space and statistical features ensures model robustness, making it adept at capturing the most vital information for classification. A long short-term memory network was used to optimize the approach, allowing precise classifications ranging from binary to five-class classification challenges, particularly fine-tuned for the Bonn Epilepsy dataset.

### Contribution

The main contributions of this work are as follows:

- This study introduces a significant advancement in epileptic seizure detection. The proposed novel deep learning method seamlessly merges the compressed latent space features from the time–frequency domain with statistical attributes of the EEG signal. This integrated feature pool captures time–frequency and statistical information, making this approach different in robustness and accuracy.
- The proposed hybrid model uses an optimal window size for EEG segmentation, ensuring minimal data loss and a set overlap ratio. After rigorous evaluation, this method selects the best window size for maximal data coverage, which is crucial for precise EEG classification. This strategy upholds data integrity, boosting the classification reliability of the model.
- A CAE was used for feature extraction from CWT images. CAEs excel at handling image data like EEG-based CWT by preserving spatial structures. The CAE retained the most important features and eliminated noise by compressing and reconstructing the image. This method reduced data dimensionality and identified the most vital EEG patterns, enhancing precision and accuracy in subsequent analysis.
- The CAE latent space features still contain some less important features. Principal Component Analysis (PCA) was applied to extract the most relevant features from the latent space, enhancing the classification accuracy.
- LSTM networks were used for classification, capitalizing on their proficiency with time-series data. Given the sequential nature of the EEG signals, LSTMs, with their ability to capture long-term dependencies, provided enhanced accuracy in detecting intricate seizure patterns.
- While many studies evaluate the Bonn dataset for binary classification, some extend to three or four classes, with few tackling a five-class problem. This study encompassed classifications from binary to five class, achieving unprecedented accuracy, i.e., 100% for binary, >95% for three and four classes, and above 93% for the five-class categorization, marking the highest recorded performance in terms of accuracy.

The remainder of this article is organized as follows. Section 2 provides an in-depth explanation of the model design and components. Section 3 reports the dataset description and the model performance on the benchmark dataset. Finally, Section 4 provides the concluding remarks on the article.

## 2. Proposed Method

**Figure 1:**
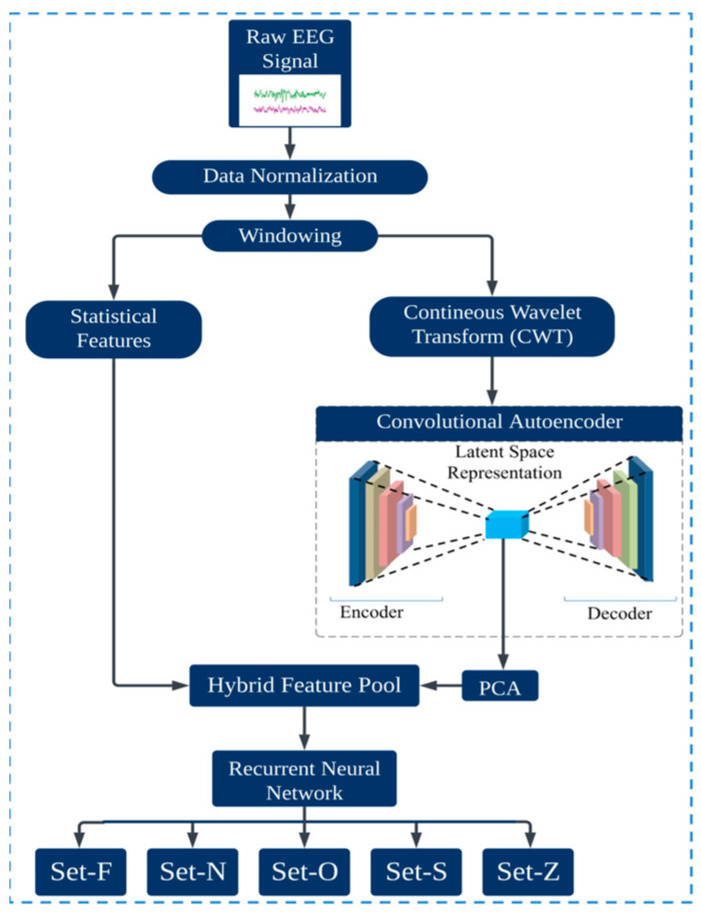
Overview of the proposed epileptic seizure detection model.

This section provides an overview of the proposed methodology for epilepsy detection, leveraging a hybrid model that combines an autoencoder and a Recurrent Neural Network (RNN), specifically the long short-term memory (LSTM) variant. The procedure starts with a windowing technique, segmenting the continuous signal into smaller, manageable packets. This approach ensures that every datum is captured accurately. Once segmented, critical statistical features for each windowed segment are calculated, capturing the primary characteristics of the data. Subsequently, the continuous wavelet transform is applied to the segmented data. This transformation extracts time–frequency information from each segment, providing a more detailed representation of the signal dynamics. The resulting time–frequency images serve as input to the Convolutional Autoencoder, which distills the data into a latent feature space. Owing to the potential high dimensionality of this latent space, PCA was implemented to streamline the feature set, retaining only those components that contribute significantly to the variance and, by extension, the classifiability of the data. These condensed features are merged with the previously computed statistical features, producing a hybrid feature pool. This comprehensive feature set captures both the inherent characteristics of the signal and its nuanced, transformed representations. Finally, this paper introduces the LSTM model, which takes this hybrid feature set as input and determines the epilepsy state of the signal. The inherent capacity of the LSTM to process sequential data makes it particularly suited for this task, ensuring accurate classifications across various detection scenarios. Figure 1 presents a visual representation of the entire process.

### 2.1. Windowing

**Figure 2:**
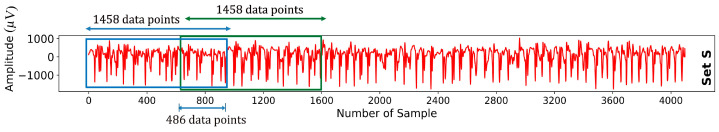
Windowing process.

The Bonn University Epilepsy dataset comprises five distinct subsets, Set Z, Set O, Set N, Set F, and Set S, and the details of which are described earlier in the introduction section. Each subset contains 100 samples, resulting in 500 samples across the entire dataset. In the present study, all 100 samples were chained, and a windowing technique was applied to create small segments of the EEG signal. In signal processing analysis, windowing plays a pivotal role, primarily in combating the challenges of spectral leakage. Spectral leakage is a key concern in signal processing, particularly relevant when analyzing EEG signals. It occurs when energy from the signal’s true frequency leaks into other frequencies, often due to the finite length of the signal window. This can distort the true frequency content of EEG data, potentially affecting the accuracy of seizure detection. Moreover, windowing enhances temporal localization, ensuring that specific spectral events are precisely mapped within distinct time frames. The technique also fine-tunes the frequency resolution, delineating closely packed frequency components with clarity [23]. Given the advantage, the sliding window technique was employed to partition each sample into multiple smaller signal segments. An overlapping sliding window method, implementing a 1458 data-point window with a 486 data-point overlap, was used to ensure no data-points were omitted. This window, shown in Figure 2, successively slides across the data, producing smaller signal segments, the combination of which represents the complete signal of the subject. The mathematical formulation of the sliding window technique with overlap, for a given signal S of length L, the starting and ending points of the $i^{th}$ windowed segment $S_{i},$ is expressed below. Equation (1) indicates the starting point of each window, and Equation (2) expresses the ending point of the window.

For the $i^{th}$ window,

(1) $$Start:S_{i}=1+(i-1)\times (\omega -\vartheta )$$

(2) $$End:S_{i}=\omega +(i-1)\times (\omega -\vartheta )$$

where;

- ω is the window length, and in this case, ω=1458.
- ϑ is the overlap length, and here, ϑ=486.
- i is the window number (e.g., i=1 for the first window, i=2 for the second, so on).
- It should be always ensured that ω>0 for the above formulation to be valid.

### 2.2. Continuous Wavelet Transformation (CWT)

Electroencephalography (EEG) records the electrical activity of the brain, producing inherently non-stationary signals. Traditional Fourier methods, which analyze the signals in terms of sinusoids with infinite duration, may not effectively capture the transient or time-varying phenomena of the EEG data [24]. On the other hand, wavelet transform is a computational method designed to analyze non-stationary signals by decomposing them into various frequency components while maintaining temporal resolution. The wavelet transform employs basic functions called “wavelets”, allowing simultaneous frequency and time domain analysis [25,26]. Equation (3) is a mathematical expression for the wavelet transform.

(3) $$WT(s,t)=\frac{1}{\sqrt{\left|s\right|}}\int_{-\infty }^{\infty }f(\tau )\psi^{*}\left(\frac{\tau -t}{s}\right)d\tau$$

where f(τ) is the input signal; $\psi^{*}(\cdot )$ represents the complex conjugate of the wavelet function; s is the scale factor (which is inversely related to frequency); and t is the translation factor (related to time).

Extending this concept, the CWT is a specialized form of wavelet transform wherein the wavelet undergoes continuous scaling and translation, allowing temporal and spectral analysis [27]. CWT’s multi-resolution characteristic is particularly advantageous for interpreting EEG signals, given that different physiological phenomena might present themselves at diverse scales. The expression for CWT of a function f(t) relative to a wavelet ψ(t) is as follows:

(4) $$CWT_{f}(s,t)=\int_{-\infty }^{\infty }f(\tau )\psi_{s,t}^{*}(\tau )d\tau$$

with the modified wavelet given by the following:

(5) $$\psi_{s,t}(\tau )=\frac{1}{\sqrt{\left|s\right|}}\psi \left(\frac{\tau -t}{s}\right)$$

- ψ is called the mother wavelet, which is a short wave-like oscillation.
- s is the scaling factor. The function is stretched if s>1 or compressed if 0<s<1.
- t is the translation factor, which shifts the function in time.
- τ is the variable of integration, typically representing time.
- The factor $\frac{1}{\sqrt{\left|s\right|}}$ is a normalization term that ensures that the wavelet has the same energy at every scale.

Equations (3) and (4) describe how the original mother wavelet, ψ, is scaled and translated to analyze a signal at various frequencies and time positions. 

**Figure 3:**
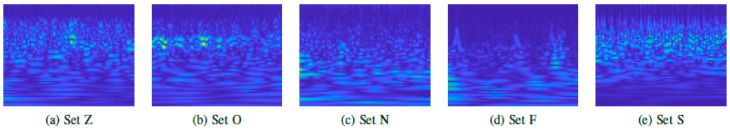
CWT images of each class.

The CWT was used to convert EEG signal segments into images, employing the Morlet wavelet. The Morlet wavelet, a complex sinusoid modulated by a Gaussian envelope, is crucial in signal processing for its ability to highlight oscillatory patterns, particularly in EEG/ECG data [28]. The CWT, with Morlet as a mother wavelet, extracted both the spectral and temporal resolutions of the signal, which were subsequently represented as images. Figure 3 shows the graphical representation of CWT images of each class of the Bonn Epilepsy dataset.

### 2.3. Convolutional Autoencoder

After being proposed by Theis et al. [29] and Balle et al. [30], the Convolutional Autoencoder (CAE) has attracted the interest of many researchers in recent years, particularly for leaned image compression. Convolutional Autoencoder is a specialized neural network that encodes and decodes data with spatial hierarchies, such as images. Unlike traditional autoencoders, CAEs utilize convolutional layers to exploit spatial localities in data, making them particularly adept at handling images. A CAE aims to approximate an identity function while adhering to specific constraints, such as limited neurons in hidden layers. A CAE is structured into two main components:

#### 2.3.1. Encoder

The encoder portion of a CAE serves as a funnel, which is responsible for mapping the input $x\in R^{n}$ to a latent (or compressed) space. This is achieved using a series of convolution operations designed to capture the spatial hierarchies in the data. Considering a feedforward neural network as the architecture, the output $h_{e}^{\left(l+1\right)}$ of the $l^{th}$ layer in the encoder is defined as follows:

(6) $$h_{e}^{\left(l+1\right)}=\sigma (W_{e}^{\left(l\right)}*h_{e}^{\left(l\right)})$$

where $W_{e}^{\left(l\right)}$ denotes the convolutional filters (or kernels), which can be considered tiny feature detectors. The nonlinear activation function, σ, introduces non-linearity into the system, allowing the network to learn complex patterns. As the EEG image progresses through the $L_{e}$ convolutional layers of the encoder, the final encoded representation, $h_{e}^{\left(L_{e}\right)}=h$, serves as a compressed, but rich, encapsulation of the most salient features of the images. 

#### 2.3.2. Decoder

The decoder acts as the inverse of the encoder. The decoder takes the compressed representation h and attempts to reconstruct it back to the original space. This involves transposed convolutional operations, which can be visualized as deconvolutions or reverse convolutions. If a feedforward neural network is considered, the output $h_{d}^{\left(l+1\right)}$ of the $l^{th}$ layer in the decoder is as follows:

(7) $$h_{d}^{\left(l+1\right)}=\sigma (W_{d}^{\left(l\right)}⊙h_{d}^{\left(l\right)})$$

where $W_{d}^{\left(l\right)}$ are the transposed convolutional filters, which operate in a manner opposite to the encoder filters. The final output from the decoder, $h_{d}^{\left(L_{d}\right)}=x\prime$, aims to be a faithful reconstruction of the original image x, bringing full circle the encoding–decoding process of the CAE.

The primary objective of a CAE is to minimize the reconstruction error between the original input and its reconstruction. This error, typically termed as the loss function, can be defined as follows:

(8) L(x′,x)=∥x′−x∥

**Figure 4:**
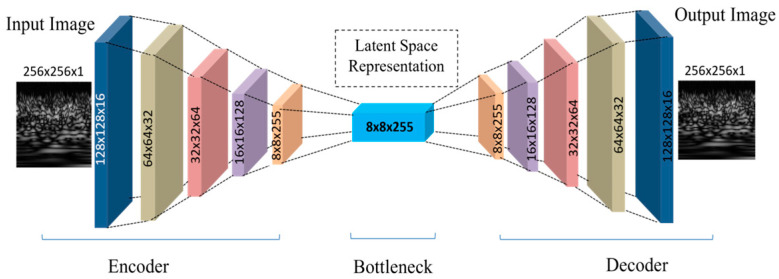
Autoencoder model architecture.

Optimization algorithms, such as backpropagation, minimize this loss when training a CAE. In the architecture presented in Table 1, a CAE was used with a five-layer encoder and decoder. The CAE’s effectiveness is demonstrated by a high PSNR value of 66 dB, indicating precise image reconstruction. Figure 4 shows the graphical layer-wise architecture of the CAE.

### 2.4. Principal Component Analysis

PCA is a well-established dimensionality reduction technique that projects data into a lower-dimensional space while preserving as much of the original variance as possible [31]. This method is particularly useful for reducing the dimensionality of datasets with many correlated variables, transforming them into a new set of orthogonal variables known as the principal components [32,33]. 

In the context of this study, PCA was used to reduce the dimensionality of the latent space extracted from the autoencoder. A compact representation of the data that retained most of the original variance was ensured by reducing the features to 128 dimensions using PCA. This processed latent space was combined with statistical features in a hybrid feature pool, paving the way for enhanced EEG signal classification.

### 2.5. Statistical Features 

Electroencephalogram (EEG) signals, which represent the electrical activities of the brain, are inherently dynamic and complex. Therefore, it is imperative to extract the representative features that capture the underlying characteristics of the EEG data to discern information from these signals, particularly for applications, such as epilepsy detection. In addition, statistical features offer a compact representation of EEG signals, distilling them into metrics that reflect the distribution and behavior of the signal over time [34]. These include the mean, standard deviation, kurtosis, skewness, and various factors, such as crest, shape, and impulse. Although each of these metrics carries its significance in capturing different signal characteristics when they provide a comprehensive overview of the signal when combined. For example, the mean offers a central tendency, suggesting the average amplitude of the signal. Standard deviation and variance capture the dispersion and variability within the signal. Metrics, such as kurtosis and skewness, provide insights into the shape of the distribution of the signal, indicating the presence of any irregular peaks or asymmetries. Factors, such as crest and shape, elucidate the transient behaviors of the signal and its oscillatory nature. Combining these statistical features with the latent features of an autoencoder derived from the CWT images can significantly enhance the classification performance of EEG signals, particularly in epilepsy detection. Because statistical features capture the basic characteristics of EEG signals, the latent space of the autoencoder, derived from the CWT images, encapsulates more complex, nonlinear patterns in the data. They offer a more comprehensive representation of the EEG signal. The fusion of these two feature sets can increase the robustness of the model. This process benefits from the generalization capabilities of autoencoders and the straightforward interpretability of statistical metrics. Furthermore, epileptic seizures lead to characteristic changes in the EEG patterns. Statistical features can highlight sudden spikes, deviations, and anomalies in the signal, which are common indicators of epileptic activities. Combined with the high-level patterns learned by the autoencoder from CWT images, the classification system can better differentiate between epileptic and non-epileptic signals. Table 2 provides the list of calculated statistical features.

### 2.6. Hybrid Features Pool

EEG signals are complex yet rich in information. It is very important to extract their right features to analyze them. With simple statistical features, a broader and more useful set of attributes can be obtained by combining the power of deep learning methods, such as CWT images. This approach combines detailed patterns (from CWT images) and basic signal traits (from statistical features) to provide a well-rounded view of the EEG data.

Ensuring the alignment of features accurately within this hybrid framework is essential to preserve data consistency and optimize subsequent analytical outcomes. $F_{AE}$ represents the set of features derived from the bottleneck of an autoencoder for a specific EEG window, and $F_{stat}$ denotes the statistical features for the same window. The harmonization of these features can be represented as follows:

(9) $$F_{hybrid_{i}}=\{f_{AE_{i}}\cup f_{stat_{i}}|f_{AE_{i}}\in F_{AE}\wedge f_{stat_{i}}\in F_{stat}\}$$

The index i in $f_{AE_{i}}$ and $f_{stat_{i}}$ ensures that the autoencoder latent space features and statistical features are obtained from the same EEG window packet. This hybrid feature pool offers a multidimensional view of EEG signals, amplifying the richness of information available in each class. This feature integration promises robustness against potential intra-class variations and maximizes the inter-class disparities, emphasizing its importance for complex data, such as EEG and EMG signal classification applications. These hybrid features are then input into an LSTM network for final classification.

### 2.7. Long Short-Term Memory

**Figure 5:**
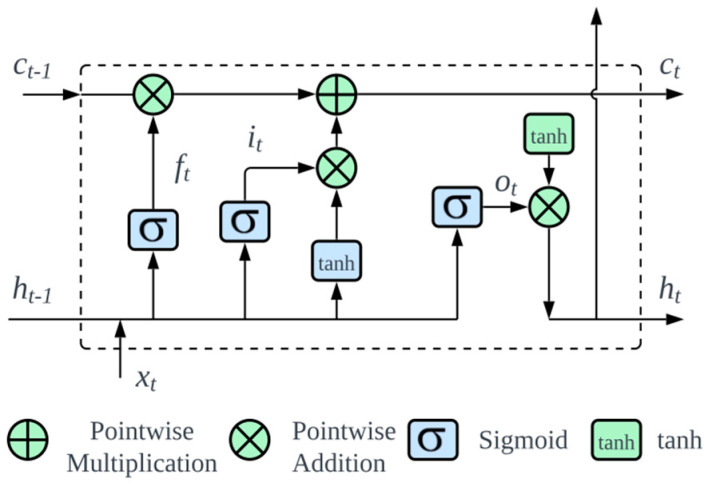
Long short-term memory unit architecture.

LSTM networks, a specific architecture of RNNs, have attracted significant attraction for predicting time-series data because of their unique cellular design. This design is essential for the LSTM to transmit information selectively, addressing issues such as vanishing and exploding gradients during backpropagation [35]. Figure 5 presents an in-depth visualization of this architecture. At the core of an LSTM are three main gates: forget, input, and output gates.

Initially, the forget gate decides the segments of information that the cell state should discard.

(10) $$f_{t}=\sigma (W_{f}\times [h_{t-1},x_{t}]+b_{f})$$

where $h_{t-1}$ denotes the prior hidden layer output; $x_{t}$ symbolizes the current input, with σ being the sigmoid activation; and W and b represent the weight matrix and bias, respectively.

Subsequently, the input gate governs the preservation of information in the cell state, spliting into identifying the data for updates and setting up an updated state. This can be expressed mathematically as follows:

(11) $$i_{t}=\sigma (W_{i}\times [h_{t-1},x_{t}]+b_{i})$$

(12) $${\tilde{C}}_{t}=tanh(W_{C}\times [h_{t-1},x_{t}]+b_{C})$$

The present state of the neuron can be derived by combining Equations (2) and (3):

(13) $$C_{t}=f_{t-1}C_{t-1}+i_{t-1}{\tilde{C}}_{t}$$

The role of the output gate is pivotal for determining the final output. The sigmoid function evaluates which segment of the cell state to assign to output, subsequently undergoing processing by the tanh function and pointwise multiplication:

(14) $$o_{t}=\sigma (W_{o}\times [h_{t-1},x_{t}]+b_{o})$$

(15) $$h_{t}=o_{t}\times tanh(C_{t})$$

In biomedical contexts, the strength of the LSTM lies in its ability to recognize the patterns over time, making it particularly effective for detecting epileptic seizures. 

EEG data, characterized by detailed time-based patterns, benefits from accuracy and timely analysis by the LSTM, ultimately improving patient care and treatment outcomes. This model uses an LSTM layer, consisting of 128 units, designed specifically to process the time-dependent patterns in EEG data. The data are passed to a dense layer using softmax activation, sorting the LSTM outputs into specific categories. The model is fine-tuned for optimal performance with the “adam” optimizer and the categorical_crossentropy loss function, which is suited for classifying multiple categories. The hyperparameters for this study were selected through a series of experiments shown in Table 3. Combining the strengths of autoencoder latent space features and statistical attributes, the LSTM provides a thorough and accurate representation of the complex patterns of the EEG data. This integration enhances the model robustness and its ability to identify subtle EEG patterns accurately, which is crucial for advanced seizure detection. The effectiveness of the proposed model will be further discussed in the next section.

## 3. Performance Evaluation

### 3.1. Meta Data

In this study, the EEG database from the University of Bonn, Germany, curated by Andrzezak et al. [36], was chosen for data incorporation. This database was selected because of its authority in the field and its frequent utilization in numerous epilepsy diagnostic studies. The dataset comprises five sets (Z, O, N, F, and S) of 100 EEG signals each, captured via a single channel from the scalp surface. Each EEG signal spans a duration of 23.6 s and includes 4097 sample points. The signals were digitized using a 12-bit A/D converter at a sampling frequency of 173.61 Hz.

**Figure 6:**
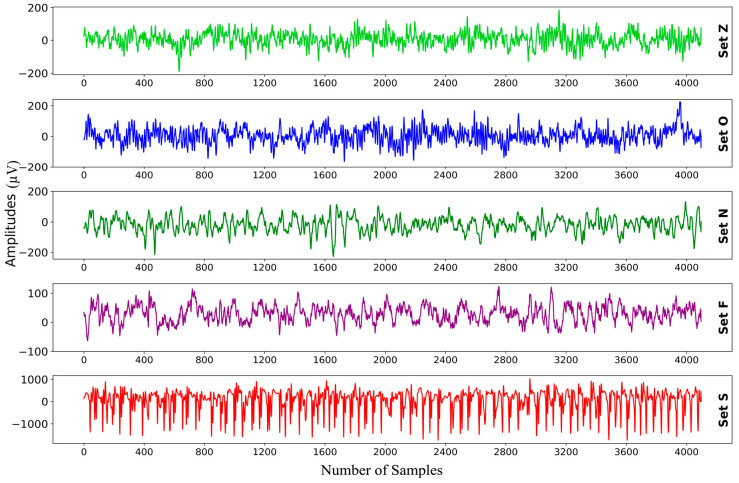
EEG signals from different classes.

In the data collection process, a total of 10 subjects were involved. Sets Z and O originate from the EEG records of five healthy individuals, with eyes open and closed, respectively. Sets N, F, and S derive from the preoperative EEG records of five diagnosed epileptic patients. In particular, Set N segments were from the hippocampus located in the opposite hemisphere of the brain. Set F was obtained from within the epileptogenic zone, with both sets containing measurements during seizure-free intervals. Set S solely encompassed the seizure activity. Table 4 provides detailed information regarding these data. For this study, all five sets were utilized, with representative EEG signal samples from each group presented in Figure 6. 

In this study, the classification performance of epilepsy seizure detection models is evaluated using multiple metrics: accuracy, F1-score, precision, recall, and sensitivity. The choice of these metrics provides a comprehensive understanding of the model proficiency in accurately identifying the seizures and distinguishing between the various classes. 

In a binary classification framework, the terminologies employed are as follows:

- True Positive (TP): instances confirmed to be positive.
- True Negative (TN): instances confirmed to be negative.
- False Positive (FP): instances incorrectly identified as positive.
- False Negative (FN): positive instances mistakenly identified as negative.

The metrics for binary classification are given by the following:

(16) $$Accuracy=\frac{TP+TN}{TP+TN+FP+FN},$$

(17) $$Precision=\frac{TP}{TP+FP},$$

(18) $$Recall\;(or\;Sensitivity)=\frac{TP}{TP+FN},$$

(19) $$F_{1}Score=2\times \frac{Precision\times Recall}{Precision+Recall}$$

In this study, the performance of the model, built upon a hybrid feature pool, was examined across different classification scenarios. The aim was to assess its proficiency in distinguishing between various numbers of classes, ranging from binary classification to a more complex five-class scenario. The specific scenarios for each classification problem are detailed as follows:

- Binary Classification: N–S, Z–S, O–S, F–S, FN–S, FNZ–S, FNO–S, and NOZ–S.
- Three-Class Classification: F–O–S, N–Z–S, O–Z–S, and FN–OZ–S.
- Four-Class Classification: F–O–Z–S and N–O–Z–S.
- Five-Class Classification: F–N–O–Z–S.

### 3.2. Binary Classification

**Figure 7:**
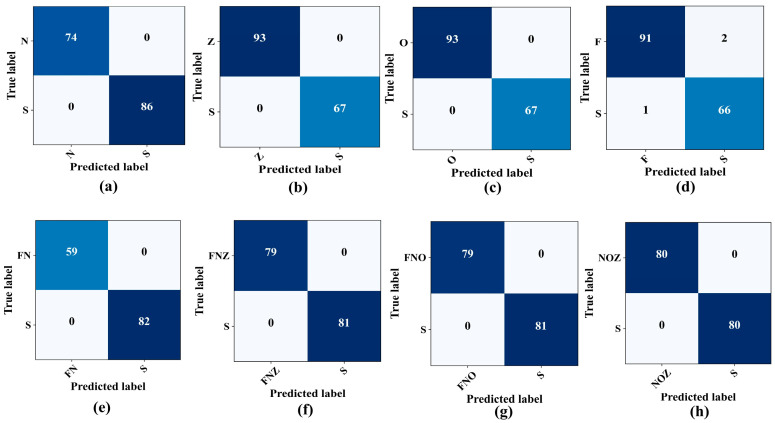
Confusion matrices for binary class classification. (**a**) N–S. (**b**) Z–S. (**c**) O–S. (**d**) F–S. (**e**) FN–S. (**f**) FNZ–S. (**g**) FNO–S. (**h**) NOZ–S.

The proposed classification system exhibited an exceptional precision in classifying critical EEG states when assessing the model performance on the previously mentioned binary cases. As highlighted in Table 5, the model differentiates between the seizure activity (Set S) and various non-seizure states, including the eye-closed (Set O), eye-open (Set Z), and seizure-free states (Sets F and N), with remarkable accuracy, often achieving accuracy and F1-scores of 100%. Nevertheless, when classifying the F–S binary combination, the model accuracy decreased slightly, settling at 98.12%. The confusion matrices, which show the true versus predicted labels across these binary combinations, are illustrated in Figure 7.

### 3.3. Three-Class Classification

**Figure 8:**
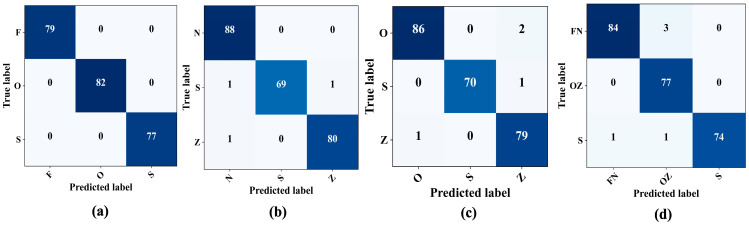
Confusion matrices for three-class classifications. (**a**) F–O–S. (**b**) N–S–Z. (**c**) O–S–Z. (**d**) FN–OZ–S.

After observing the promising results from the model performance for binary class problems, the tests were extended to multi-class problems, specifically F–O–S, N–Z–S, O–Z–S, and FN–OZ–S. The initial approach involved classifying three distinct categories: the normal state, characterized by patients with closed eyes (Class “O”); the interictal state, representing patients diagnosed with epilepsy but currently in a seizure-free state (Class F); and the ictal state, indicative of active seizures. The proposed epilepsy seizure detection architecture classified these three states, achieving 100% accuracy with no misclassifications, as shown in Figure 8a. Furthermore, another set of three-class classification problems, the N–Z–S classification problem, evaluated the model performance. The confusion matrix in Figure 8b shows that the model precision remained high, achieving an overall accuracy and sensitivity of 98.75% and 97.2%, respectively, for detecting seizures. This performance was consistent, with an F1-score and a precision rate of 98.76%. In the subsequent O–S–Z and FN–OZ–S classifications, the model sustained its robust performance, surpassing the accuracy and sensitivity of 96% and 98%, respectively, for seizure detection (Figure 8c,d). Table 6 lists the comprehensive performance of the proposed model for different three-class problems.

### 3.4. Four-Class Classification

**Figure 9:**
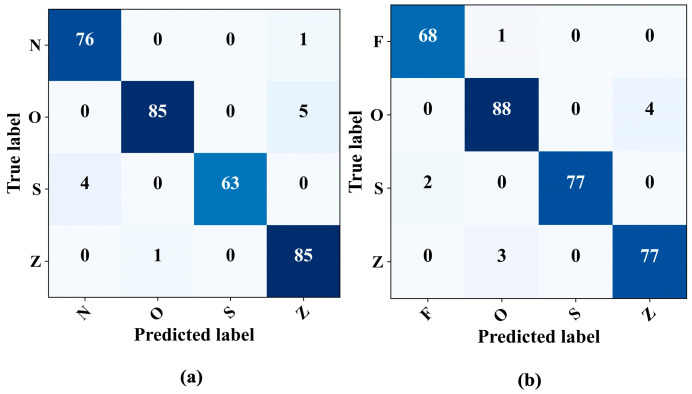
Confusion matrices for four-class classification. (**a**) N–O–S–Z. (**b**) F–O–S–Z.

The model’s capabilities for detecting epileptic EEG signals in four-class problems were assessed thoroughly. In particular, two different scenarios were examined: the N–O–Z–S and F–O–S–Z classifications. In both cases, the model showed high performance even in four-class problems, as illustrated in the confusion matrices in Figure 9. The classification consistently achieved an approximate accuracy and precision of 97%. Table 7 provides a detailed overview of the model metrics for these four-class classification problems. 

### 3.5. Five-Class Classification

**Figure 10:**
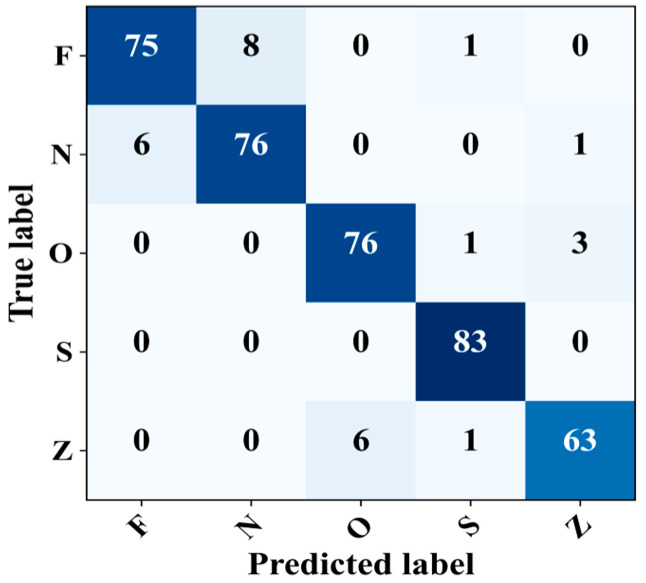
Confusion matrix for five-class classification.

Finally, the proposed model was evaluated for its ability to detect epileptic EEG samples within complex signals. The model’s performance was evaluated using the Z–N–O–Z–S five-class problem. The confusion matrix shows that the model achieved promising results with an overall accuracy, F1-score, precision, and general sensitivity of 93.25%, 93.21%, 93.23%, and 93.25%, respectively, as shown in Figure 10. In particular, the model revealed a sensitivity of 100% in detecting the epileptic seizure signals with no false detection. The model also recorded a sensitivity of 95.00%, 91.56%, and 90% for class O, class N, and classes Z and F, respectively. In summary, these results confirm the reliable detection performance of the model across various scenarios, i.e., binary, three-class, four-class, or even five-class problems. 

## 4. Discussion

After evaluating the model across various classification problems, ranging from binary to three-class, four-class, and even five-class scenarios, we observed that the proposed algorithm showed promising results in all these tasks. The enhanced performance of our epilepsy detection model is due to its hybrid architecture. This hybrid design leverages the autoencoder’s feature distillation from high-dimensional data and the LSTM’s sequential information processing. The integration of PCA retains key classification components, and merging these with statistical features creates a comprehensive feature set. This fusion effectively captures diverse signal characteristics, enhancing data classifiability. To assess the impact of concatenating statistical features with CAE (Convolutional Autoencoder) latent space features, we conducted an ablation study within a five-class classification framework. Table 8 illustrates the outcomes of training the LSTM network with distinct feature sets. When solely CAE latent space features were used, the LSTM achieved an accuracy of 89.50%, an F-1 score of 89.57%, a precision of 89.83%, and a sensitivity for the epileptic class of 91.78%. In contrast, training with only statistical features resulted in lower performance across all metrics, with an accuracy of 78.50%, an F-1 score of 78.60%, a precision of 79.17%, and a sensitivity for the epileptic class of 82.19%. However, the combination of both CAE latent space features and statistical features substantially improved the model’s performance, elevating the accuracy to 93.25%, F-1 score to 93.21%, and precision to 93.23%, and achieving a perfect sensitivity for the epileptic class at 100%. This demonstrates that the integration of both feature types significantly enhances the LSTM network’s ability to classify and detect epilepsy in a multi-class setting. The LSTM’s proficiency in sequential data analysis further ensures accurate epilepsy detection across various scenarios. Overall, our approach sets a new standard in EEG data analysis for epilepsy detection. The performance of the proposed model was compared with existing approaches. Table 9 shows a comparison of the proposed model with some existing approaches.

## 5. Conclusions

This paper introduced an advanced intelligent EEG recognition framework for epileptic seizure detection. This framework integrates deep autoencoders, statistical features, and LSTM networks. An optimal overlapping windowing method was used to mitigate the inherent spectral leakage. Subsequently, the CWT was used to produce time–frequency images from each window. Simultaneously, the statistical attributes, such as mean, mode, and standard deviation, were extracted during this wavelet transformation. A deep convolutional autoencoder (CAE) was trained to extract the essential features from the CWT images. The latent space of this CAE, rich with features, was then refined using PCA and concatenated with the statistical features, forming a comprehensive hybrid feature pool. This enhanced pool was processed through LSTM-based classification, addressing multiple class problems.

The model demonstrated exceptional F-1 score, precision, and accuracy. In most cases, it exhibited error-free classification in binary class problems, while in three- and four-class problems, it exhibited over 95% and 93% accuracy, respectively. The model sensitivity metrics are equally notable, scoring 100% for binary and some three-class situations, maintaining over 97% for all three-class problems, and >94% for four-class problems. Averaging across all classifications, this model achieved an accuracy exceeding 97%, highlighting its stability and validating its ability to detect epileptic events accurately within complex signal scenarios.

## Figures and Tables

**Table 1:** Summary of the autoencoder architecture.

**Encoder**		
**Layer (type)**	**Output Shape**	**Param#**
Conv2D	(None, 128, 128, 16)	160
Conv2D	(None, 64, 64, 32)	4640
Conv2D	(None, 32, 32, 64)	18,496
Conv2D	(None, 16, 16, 128)	73,856
Conv2D	(None, 8, 8, 255)	294,015
**Total parameters**	391,167	
**Trainable parameters**	391,167	
**Non-trainable parameters**	0	
**Decoder**		
**Layer (type)**	**Output Shape**	**Param#**
Conv2D Transpose	(None, 16, 16, 128)	293,888
Conv2D Transpose	(None, 32, 32, 64)	73,792
Conv2D Transpose	(None, 64, 64, 32)	18,464
Conv2D Transpose	(None, 128, 128, 16)	4624
Conv2D Transpose	(None, 256, 256, 1)	145
**Total parameters**	390,913	
**Trainable parameters**	390,913	
**Non- trainable parameters**	0	

**Table 2:** Statistical features and their mathematical expressions.

Feature	Mathematical Expression	Feature	Mathematical Expression
Minimum	min(s)	Range	max(s)−min(s)
Maximum	max(s)	Energy	$\sum s^{2}$
Mean	$\frac{1}{N}\sum s$	Clearance Factor	$\frac{max(|s|)}{\sqrt{\frac{1}{N}\sum |s{|}^{2}}}$
Standard Deviation	$\sqrt{\frac{1}{N-1}\sum (s-\mu {)}^{2}}$	Variance	$\frac{1}{N-1}\sum (s-\mu {)}^{2}$
Kurtosis	$\frac{\frac{1}{N}\sum (s-\mu {)}^{4}}{\sigma^{4}}$	Impulse Factor	$\frac{max(|s|)}{\frac{1}{N}\sum |s|}$
Skewness	$\frac{\frac{1}{N}\sum (s-\mu {)}^{3}}{\sigma^{3}}$	Power	$\frac{\sum s^{2}}{N}$
RMS	$\sqrt{\frac{1}{N}\sum s^{2}}$	Peak to RMS	$\frac{max(|s|)}{\sqrt{\frac{1}{N}\sum s^{2}}}$
Crest Factor	$\frac{max(s)}{\sqrt{\frac{1}{N}\sum s^{2}}}$	Shape Factor	$\frac{\sqrt{\frac{1}{N}\sum s^{2}}}{\frac{1}{N}\sum |s|}$

**Table 3:** Hyperparameters Tuning.

Hyperparameter	Fixed Parameters	Values Tested	Accuracies (%)
Number of Neurons	Epochs = 50, Batch Size = 32	32, 64, 128, 256	90.28, 91.65, 93.25, 92.78
Batch Size	Neurons = 128, Epochs = 50	16, 32, 64, 128	93.06, 92.22, 93.30, 92.36
Number of Epochs	Neurons = 128, Batch Size = 32	20, 30, 40, 50	86.25, 89.40, 92.30, 93.35

**Table 4:** Overview of EEG Bonn EEG dataset of University of Bonn, Germany.

	Patient Stage	Subject Activities	Number of Samples	Length of Segments	Sampling Frequency (Hz)	Duration (s)
Epileptic	Ictal	Set S (Seizure Activity)	100	4097	173.61	23.60
	Interictal	Set F (Seizure Free)	100	4097	173.61	23.60
		Set N (Seizure Free)	100	4097	173.61	23.60
Healthy	Normal	Set O (Eyes Closed)	100	4097	173.61	23.60
		Set Z (Eyes Open)	100	4097	173.61	23.60

**Table 5:** Performance metrics for binary classification.

Problem	Accuracy (%)	F1-Score (%)	Precision (%)	Sensitivity (%)
N–S	100	100	100	Class N: 100 Class S: 100
Z–S	100	100	100	Class Z: 100 Class S: 100
O–S	100	100	100	Class O: 100 Class S: 100
FN–S	100	100	100	Class FN:100 Class S: 100
FNZ–S	100	100	100	Class FNZ: 100 Class S: 100
FNO–S	100	100	100	Class FNO: 100 Class S: 100
NOZ–S	100	100	100	Class NOZ: 100 Class S: 100
F–S	98.12	98.12	98.13	Class FNZ: 97.85 Class S: 98.5

**Table 6:** Performance metrics for the three-class classification.

Problem	Accuracy (%)	F1-Score (%)	Precision (%)	Sensitivity (%)
F–O–S	100	100	100	Class F: 100 Class O: 100 Class S: 100
N–Z–S	98.75	98.75	98.76	Class Z: 98.76 Class N: 100 Class S: 97.2
O–Z–S	96.25	96.26	96.37	Class O: 93.18 Class Z: 98.60 Class S: 97.53
FN–OZ–S	98	97.93	97.98	Class FN: 96.56 Class OZ: 100 Class S: 97.40

**Table 7:** Performance metrics for four-class classification.

Problem	Accuracy (%)	F1-Score (%)	Precision (%)	Sensitivity (%)
N–O–Z–S	96.60	96.57	96.70	Class N: 98.72 Class O: 94.51 Class S: 94.03 Class Z: 98.84
F–O–Z–S	98.75	98.75	98.76	Class F: 98.56 Class O: 95.65 Class S: 97.50 Class Z: 96.25

**Table 8:** Ablation experiment.

Training Features	Test Accuracy (%)	F-1 Score (%)	Precision (%)	Sensitivity for Epileptic Class (%)
CAE latent space features	89.50	89.57	89.83	91.78
Statistical features	78.50	78.60	79.17	82.19
Combined features	93.25	93.21	93.23	100

**Table 9:** Comparison with some existing approaches.

Author	Year	Method Used	Classifier	Classification Problem	Results
Zarei et al. [37]	2021	DWT	SVM	Z–S, O–S N–S, F–S	99.50, 99.75 99.00, 99.50
Wang et al. [38]	2019	Symlets wavelets and PCA	SVM	Z–S, O–S N–S, F–S	100 98.4, 98.1
Yazid et al. [11]	2023	DWT, local binary pattern transition histogram, and local binary pattern mean absolute deviation	KNN	Z–S O–S N–S F–S	99.94 99.86 99.88 99.70
Gupta et al. [18]	2019	Fourier Bassel series expansion and weighted multi-scale Renyi permutation entropy	LS-SVM	Z–S O–S N–S F–S	99.50 99.50 99.50 97.50
Mamli et al. [39]	2019	Fourier Synchro-Squeezed Transform and gray level co-occurrence matrix	KNN, SVM	ZO–S FN–S	99.73 99.59
Mandhouj et al. [26]	2021	STFT spectograms	CNN	ZO–S	98.33
Bari et al. [40]	2020	EMD with normalized intrinsic mode function	Quadratic Discriminant Analysis (QDA)	NF–S	99.00
Kaur et al. [41]	2023	Activations from conv5	SVM	ZNF–S Z–N–S	99.75 98.00
Zhao et al. [42]	2019	Stationary WT and entropy features	Back-Propagation NN	ZO–NF–S	93.30
Baykara et al. [43]	2021	Stockwell Transform, Entropies, and Perservals energy	ELM	ZO–NF–S	90.00
Turk et al. [44]	2019	FFT, STFT, WT Transform	CNN	Z–N–F–S O–N–F–S Z–O–N–F	90.50 91.50 93.60
Zhang et al. [45]	2021	Frequency Slice WT (FSWT), Fuzzy entropy, and Higuchi FD	t-distributed stochastic neighbor embedding (t-SNE)	Z–O–N–F–S	93.62
Zhou et al. [46]	2020	DWT entropy features	RBF NN	Z–O–N–F–S	78.40
				N–S, Z–S, O–S	100
				FN–S, FNZ–S,	
				FNO–S, NOZ–S	100
				F–S	98.12
				F–O–S	100
This Proposed Study		CWT and statistical features	LSTM	N–Z–S	98.75
Bonn Epilepsy dataset				O–Z–S	96.25
				FN–OZ–S	98.00
				N–O–Z–S	96.60
				F–O–Z–S	97.00
				F–N–O–Z–S	93.25
This Proposed Study					
CHB-MIT-Epilepsy dataset		CWT, Statistical Features	LSTM	Ictal-interictal	96.45

## Data Availability

The datasets that support the findings of this study are openly available and are as follows. Ralph G. Andrzejak1, Klaus Lehnertz1, Florian Mormann, Christoph Rieke1, Peter David, and Christian E. Elger1, “Bonn Epilepsy Dataset”. [Online]. Available: https://www.ukbonn.de/epileptologie/arbeitsgruppen/ag-lehnertz-neurophysik/downloads/ (Accessed on 3 March 2023).

## References

[1] Kuhlmann L., Lehnertz K., Richardson M.P., Schelter B., Zaveri H.P. (2018). Seizure prediction—Ready for a new era. Nat. Rev. Neurol..

[2] Liu T., Shah M.Z.H., Yan X., Yang D. (2023). Unsupervised feature representation based on deep boltzmann machine for seizure detection. IEEE Trans. Neural Syst. Rehabil. Eng..

[3] Rungratsameetaweemana N., Lainscsek C., Cash S.S., Garcia J.O., Sejnowski T.J., Bansal K. (2021). Brain network dynamics codify heterogeneity in seizure propagation. bioRxiv.

[4] Larivière S., Rodríguez-Cruces R., Royer J., Caligiuri M.E., Gambardella A., Concha L., Keller S.S., Cendes F., Yasuda C., Bonilha L. (2020). Network-based atrophy modeling in the common epilepsies: A worldwide ENIGMA study. Sci. Adv..

[5] Ahmad I., Wang X., Javeed D., Kumar P., Samuel O.W., Chen S. (2023). A hybrid deep learning approach for epileptic seizure detection in eeg signals. IEEE J. Biomed. Health Inform..

[6] Bomela W., Wang S., Chou C.-A., Li J.-S. (2020). Real-time Inference and Detection of Disruptive EEG Networks for Epileptic Seizures. Sci. Rep..

[7] Zhu G., Li Y., Wen P., Wang S. (2015). Classifying epileptic eeg signals with delay permutation entropy and multi-scale k-means. Signal and Image Analysis for Biomedical and Life Sciences.

[8] Raeisi K., Khazaei M., Croce P., Tamburro G., Comani S., Zappasodi F. (2022). A graph convolutional neural network for the automated detection of seizures in the neonatal EEG. Comput. Methods Programs Biomed..

[9] Akyol K. (2020). Stacking ensemble based deep neural networks modeling for effective epileptic seizure detection. Expert Syst. Appl..

[10] da Silva Lourenc C., Tjepkema-Cloostermans M.C., van Putten M.J. (2021). Machine learning for detection of interictal epileptiform dis- charges. Clin. Neurophysiol..

[11] Yazid M., Fahmi F., Sutanto E., Shalannanda W., Shoalihin R., Horng G.-J., Aripriharta (2021). Simple detection of epilepsy from eeg signal using local binary pattern transition histogram. IEEE Access.

[12] Malekzadeh A., Zare A., Yaghoobi M., Kobravi H.-R., Alizadehsani R. (2021). Epileptic seizures detection in eeg signals using fusion handcrafted and deep learning features. Sensors.

[13] Aayesha, Afzaal M., Qureshi M.S., Fayaz M. (2021). Machine learning-based EEG signals classification model for epileptic seizure detection. Multimed. Tools Appl..

[14] Sharmila A., Geethanjali P. (2016). DWT based detection of epileptic seizure from eeg signals using naive bayes and k-nn classifiers. IEEE Access.

[15] Al-Hadeethi H., Abdulla S., Diykh M., Deo R.C., Green J.H. (2020). Adaptive boost ls-svm classification approach for time-series signal classification in epileptic seizure diagnosis applications. Expert Syst. Appl..

[16] Beeraka S.M., Kumar A., Sameer M., Ghosh S., Gupta B. (2021). Accuracy enhancement of epileptic seizure detection: A deep learning approach with hardware realization of stft. Circuits Syst. Signal Process..

[17] Driscoll N., Rosch R.E., Murphy B.B., Ashourvan A., Vishnubhotla R., Dickens O.O., Johnson A.T.C., Davis K.A., Litt B., Bassett D.S. (2021). Multimodal in vivo recording using transparent graphene microelectrodes illuminates spatiotemporal seizure dynamics at the microscale. Commun. Biol..

[18] Omidvar M., Zahedi A., Bakhshi H. (2021). Eeg signal processing for epilepsy seizure detection using 5-level db4 discrete wavelet transform, ga-based feature selection and ann/svm classifiers. J. Ambient. Intell. Humaniz. Comput..

[19] Gupta V., Pachori R.B. (2019). Epileptic seizure identification using entropy of fbse based eeg rhythms. Biomed. Signal Process. Control.

[20] Na J., Wang Z., Lv S., Xu Z. (2021). An extended k nearest neighbors-based classifier for epilepsy diagnosis. IEEE Access.

[21] Polat K., Nour M. (2020). Epileptic seizure detection based on new hybrid models with electroen-cephalogram signals. IRBM.

[22] Miltiadous A., Tzimourta K.D., Giannakeas N., Tsipouras M.G., Glavas E., Kalafatakis K., Tzallas A.T. (2023). Machine learning al- gorithms for epilepsy detection based on published eeg databases: A systematic review. IEEE Access.

[23] Piho L., Tjahjadi T. (2020). A mutual information based adaptive windowing of informative eeg for emotion recognition. IEEE Trans. Affect. Comput..

[24] Yang X., Zhao J., Sun Q., Lu J., Ma X. (2021). An effective dual self-attention residual network for seizure prediction. IEEE Trans. Neural Syst. Rehabil. Eng..

[25] Shankar A., Dandapat S., Barma S. (2022). Seizure types classification by generating input images with in-depth features from decomposed eeg signals for deep learning pipeline. IEEE J. Biomed. Health Inform..

[26] Humairani A., Rizal A., Wijayanto I., Hadiyoso S., Fuadah Y.N. Wavelet-based entropy analysis on eeg signal for detecting seizures. Proceedings of the 2022 10th International Conference on Information and Communication Technology (ICoICT).

[27] Shuvo S.B., Ali S.N., Swapnil S.I., Hasan T., Bhuiyan M.I.H. (2021). A lightweight cnn model for detecting respiratory diseases from lung auscultation sounds using emd-cwt-based hybrid scalogram. IEEE J. Biomed. Health Inform..

[28] Bu R. (2007). An algorithm for the continuous morlet wavelet transform. Mech. Syst. Signal Process..

[29] Theis L., Shi W., Cunningham A., Husza F. (2017). Lossy image compression with compressive autoencoders. arXiv.

[30] Balle J., Laparra V., Simoncelli E.P. (2016). End-to-end optimized image compression. arXiv.

[31] Metzner C., Schilling A., Traxdorf M., Schulze H., Tziridis K., Krauss P. (2023). Extracting continuous sleep depth from EEG data without machine learning. Neurobiol. Sleep Circadian Rhythm..

[32] Ashraf M., Anowar F., Setu J.H., Chowdhury A.I., Ahmed E., Islam A., Al-Mamun A. (2023). A survey on dimensionality reduction techniques for time-series data. IEEE Access.

[33] Ataee P., Yazdani A., Setarehdan S.K., Noubari H.A. (2007). Manifold learning applied on eeg signal of the epileptic patients for detection of normal and pre-seizure states. Proceedings of the 2007 29th Annual International Conference of the IEEE Engineering in Medicine and Biology Society.

[34] Gu X., Cao Z., Jolfaei A., Xu P., Wu D., Jung T.P., Lin C.T. (2021). EEG-based brain-computer interfaces (BCIs): A survey of recent studies on signal sensing technologies and computational intelligence approaches and their applications. IEEE/ACM Trans. Comput. Biol. Bioinform..

[35] Rabby M.K.M., Eshun R.B., Belkasim S., Islam A.K. Epileptic seizure detection using eeg signal based lstm models. Proceedings of the 2021 IEEE Fourth International Conference on Artificial Intelligence and Knowl- edge Engineering (AIKE).

[36] Andrzejak R.G., Lehnertz K., Mormann F., Rieke C., David P., Elger C.E. (2001). Indications of nonlinear deterministic and finite-dimensional structures in time series of brain electrical activity: Dependence on recording region and brain state. Phys. Rev. E.

[37] Zarei A., Asl B.M. (2021). Automatic seizure detection using orthogonal matching pursuit, discrete wavelet transform, and entropy based features of eeg signals. Comput. Biol. Med..

[38] Wang X., Gong G., Li N. (2019). Automated recognition of epileptic eeg states using a combination of symlet wavelet processing, gradient boosting machine, and grid search optimizer. Sensors.

[39] Mamli S., Kalbkhani H. (2019). Gray-level co-occurrence matrix of fourier synchro-squeezed transform for epileptic seizure detection. Biocybern. Biomed. Eng..

[40] Bari M.F., Fattah S.A. (2020). Epileptic seizure detection in eeg signals using normalized imfs in ceemdan domain and quadratic discrimi- nant classifier. Biomed. Signal Process. Control.

[41] Kaur T., Gandhi T.K. (2023). Automated diagnosis of epileptic seizures using eeg image representations and deep learning. Neurosci. Inform..

[42] Zhao X., Zhang R., Mei Z., Chen C., Chen W. (2019). Identification of epileptic seizures by characterizing instantaneous energy behavior of eeg. IEEE Access.

[43] Baykara M., Abdulrahman A. (2021). Seizure detection based on adaptive feature extraction by applying extreme learning machines. Trait. Signal.

[44] Türk Ö., Özerdem M.S. (2019). Epilepsy detection by using scalogram based convolutional neural network from eeg signals. Brain Sci..

[45] Zhang T., Han Z., Chen X., Chen W. (2021). Subbands and cumulative sum of subbands based nonlinear features enhance the performance of epileptic seizure detection. Biomed. Signal Process. Control.

[46] Zhou D., Li X. (2020). Epilepsy eeg signal classification algorithm based on improved rbf. Front. Neurosci..

